# High-resolution analysis of recent population structure using rare variants

**DOI:** 10.1093/g3journal/jkag100

**Published:** 2026-04-24

**Authors:** Lei Huang, Thiseas C Lamnidis, Stephan Schiffels

**Affiliations:** Department of Archaeogenetics, Max Planck Institute for Evolutionary Anthropology, Leipzig 04103, Germany; Department of Archaeogenetics, Max Planck Institute for Evolutionary Anthropology, Leipzig 04103, Germany; Department of Archaeogenetics, Max Planck Institute for Evolutionary Anthropology, Leipzig 04103, Germany

**Keywords:** population structure, rare variation, ancient DNA

## Abstract

Identifying population structure from genetic data is a key challenge, for which several statistical methods have been developed, including *F*-statistics, which measure the average correlation in allele frequency differences between two pairs of populations. *F*-statistics are typically applied to a subset of genetic variation within the common allele frequency band, available through microarrays and SNP enrichment techniques. Recent advances in sequencing technology increasingly allow generating whole-genome sequencing data, both ancient and modern, which not only enable querying nearly every base of the genome, but also contain numerous rare variants. Rare variants, with their more population-specific distribution, allow detection of recent population structure with much finer resolution than common variants - an opportunity that has so far been under-exploited. Here, we develop a new statistical method, RAS (Rare Allele Sharing), for summarizing rare allele frequency correlations, similar to *F*-statistics but with flexible ascertainment on allele frequencies. We test RAS on both published and simulated data and find that RAS, with appropriate ascertainment, has better resolution than genome-wide *F*-statistics in identifying population structure caused by recent demographic events. Leveraging this, we further develop the use of RAS to compute ancestry proportions accurately in cases of recently diverged and closely-related source populations. We implemented the new statistical methods as an R package and a command line tool. In summary, our method can provide new perspectives to identify and model population structure, allowing us to understand more subtle relationships among populations in the recent human past.

## Introduction

Human population structure is shaped by past demographic events, which in turn can be inferred using genomic data. For example, populations isolated from each other for an extended period of time will differ in their allele frequencies due to genetic drift (exacerbated in case of small population sizes). On the other hand, migrations and admixture tend to equalize allele frequencies and affect population structure. Therefore, by analyzing and modeling population structure we can infer demographic processes. To identify population structure and make inferences on past demographic events (such as isolation and migration), a variety of statistical methods have been developed and established in the field.

One popular approach is *F*-statistics (Patterson et al. 2012; Peter 2016), subdivided into $F_{2}$, $F_{3}$ and $F_{4}$ depending on the number of populations involved. All *F*-statistics can be formulated as $F_{4}$, and therefore measure the average covariance in allele frequency differences between two pairs of populations (Reich et al. 2009; Lipson 2020), and reflect the overlap between two genetic drift paths in a demographic model of the relationships of all populations involved (Reich et al. 2009; Patterson et al. 2012). *F*-statistics were first proposed in Reich et al. (2009) to test for “tree-ness” and compute the admixture proportion of focal populations rejecting tree-ness. *F*-statistics have been widely applied in human archaeogenetics, such as testing for genetic similarity of populations (Raghavan et al. 2014), determining ancestry components (Reich et al. 2012; Lazaridis et al. 2014) and detecting past admixture events (Patterson et al. 2012).

One critical advantage of *F*-statistics, unlike many other methods relying on allele frequency modeling (e.g. momi Kamm et al. 2020, fastsimcoal Excoffier et al. 2013, dadi Gutenkunst et al. 2009), is their robustness to some forms of SNP ascertainment. Specifically, it was shown theoretically (and in simulations) that statistical tests based on *F*-statistics (e.g. for tests for admixture) are unbiased under certain outgroup-directed ascertainment schemes (Patterson et al. 2012). It turns out, empirically, that non-outgroup-directed ascertainments are also close to being unbiased (although see Flegontov et al. 2023). This robustness rendered *F*-Statistics to be the ideal tool for SNP-ascertained datasets (e.g. Illumina 650 K Li et al. 2008, Affymetrix Human Origins Patterson et al. 2012, 1240K Mathieson et al. 2015), which for a long time have been the primary tool for obtaining genome-wide variation (Li et al. 2008; Novembre and Stephens 2008; Patterson et al. 2012).

However, in recent years sequencing costs have dropped and large amounts of whole-genome sequencing datasets have been generated, certainly for modern DNA (The 1000 Genomes Project 2015; Bergström et al. 2020) but increasingly also for ancient DNA (Allentoft et al. 2015; Orlando et al. 2021; Hui et al. 2024; McColl et al. 2025a, 2025b). This shift to shotgun data enables new opportunities to move beyond random subset SNP ascertainment used with capture enrichment and genotyping technologies. Shotgun sequencing makes it possible to querying nearly every base of the genome, allowing the application of advanced demographic inference methods, as well as addressing potential bias in *F*-statistics of samples genotyped via SNP arrays or in-solution target capture (Flegontov et al. 2023). More importantly, whole-genome sequencing data contain many rare variants (The 1000 Genomes Project 2015; Bergström et al. 2020), which are more likely to be recently derived and can lead to novel conclusions on recent demographic history. An example is the phylogeny between Central African Mbuti, West African Yoruba and East Asian Han. From genome-wide variation, we infer Mbuti to be an outgroup with the phylogeny (Mbuti, (Yoruba, Han)), reflecting deep ancestral population structure inside of Africa. This phylogeny results in a significantly negative genome-wide statistic *D*(Chimp, Yoruba; Han, Mbuti) (Bergström et al. 2020). However, when restricting to variants of low derived allele frequency in Yoruba, this statistic becomes significantly positive, suggesting recent gene flow between Mbuti and Yoruba and contradicting the genome-wide phylogeny, showing how rare variation can emphasize recent demographic history.

Rare variation has been recognized as a potential tool for identifying fine-scale population structure, especially for distinguishing closely related populations. When quantifying the affinity between pairs of individuals in the 1,000 Genomes Project with doubleton sharing, rather than genotype covariance, boundaries among populations are more pronounced, and even subgroups in populations GBR (from Britain) and CHS (from Southern China) can be detected. That is because many more doubletons are shared within the same subgroup than between different subgroups of the same population (The 1000 Genomes Project 2015). Rare allele methods have also been successfully applied to ancestry estimation. When focusing on alleles with lower frequency, ancient British populations from the Iron Age and Anglo-Saxon period are better distinguished by the ratio of alleles they share with Dutch and Spanish, therefore allowing accurate estimation on the Anglo-Saxon British ancestry in present-day British populations (Schiffels et al. 2016). Similarly, among North American indigenous groups, present-day Athabaskans can be distinguished from other groups due to recent admixture from Paleo-Eskimos, and therefore can be modeled as being admixed between northern First Peoples and Paleo-Eskimos (Flegontov et al. 2019).

However, rare allele methods have not yet been coherently formalized in earlier publications. Considering the similarity between *F*-statistics and previous rare allele methods, in this article we define RAS-statistics for rare variation, and demonstrate through simulation and empirical data their ability to outperform ordinary *F*-statistics in detecting recent demographic events, even when the latter are applied to whole-genome data. We derive a RAS-based method for ancestry decomposition and show with simulations that it gives more accurate estimates than *F*-Statistics based ancestry proportions in cases of recent population structure between the sources.

## Method

### RAS : Rare allele sharing statistics

Here, we define a statistic summarizing rare allele frequency correlations, RAS, an acronym for “Rare Allele Sharing”. RAS-statistics are computed on genome-wide biallelic SNPs, similar to *F*-statistics (Patterson et al. 2012) but with ascertainment on rare variants.

In order to define RAS-statistics, we first define the following concepts:

**Reference population**  *R* A group of individuals that is used to ascertain variants within specific allele frequency ranges.

**Outgroup**  *O* An individual or group, which is considered an outgroup to all other individuals/groups involved in the analysis. It is used to define the ancestral allele, and hence polarize alleles into ancestral and derived.

**Genome length**  *L* The number of all positions in the genome considered for analysis. Usually this covers all biallelic SNPs in large panels such as 1,000 Genomes (The 1000 Genomes Project 2015) and/or HGDP (Bergström et al. 2020).

**Sample allele frequencies**  $x_{A}$ A vector of length *L* representing outgroup-directed derived allele frequencies in population *A*. We use the expression $x_{A,i}$ to refer to sample allele frequency *A* at position *i*.

**Ascertained SNP set**  $M(O,R,f_{\min},f_{\max})$ The set of all positions at which i) the outgroup is homozygous for the reference allele, and ii) the derived allele frequency (polarized via outgroup *O*) in reference population *R* is between $f_{\min}$ and $f_{\max}$.

**Ascertained non-missing overlap**  $L_{M}(A,B)$ the number of sites in *M* that are non-missing in populations *A* and *B*, with both populations possessing at least one individual with available genotype.

We then define a simple statistic as the correlation of two frequencies (for brevity, we write *M* instead of $M(O,R,f_{\min},f_{\max})$):


(1) $$RAS(A;B)=\;\frac{1}{L_{M}(A,B)}x_{A}^{T}x_{B}=\frac{1}{L_{M}(A,B)}\sum_{i\in M}x_{A,i}x_{B,i}$$

Intuitively, this statistic measures the average rate of allele sharing among any pair of haplotypes from groups *A* and *B* across all ascertained variants.

Indeed, there is a close correspondence of RAS(A;B) and outgroup-$F_{3}$-statistics, which are more generally defined as


$$F_{3}(A,B;O)=\frac{1}{L}(x_{O}-x_{A}{)}^{T}(x_{O}-x_{B}).$$

Using derived allele frequencies polarized by our ascertainment outgroup *O*, and using only monomorphic sites in *O*, this definition simplifies to $x_{A}^{T}x_{B}$ as in equation (1). While *RAS* is mathematically derived from $F_{3}$-statistics, to avoid cumbersome extensions in notation and ambiguity in interpretation, we use the term *RAS* to denote this specific conditional framework.

From our basic form of RAS-Statistics (equation (1)), we derive the following RAS-differences, termed RASD:


(2) $$\begin{array}{cc} RASD(A_{1},A_{2};B) & =\;RAS(A_{1};B)-RAS(A_{2};B)\\ RASD(A;B_{1},B_{2}) & =\;RAS(A;B_{1})-RAS(A;B_{2})\\ RASD(A_{1},A_{2};B_{1},B_{2}) & =\;RASD(A_{1};B_{1},B_{2})-RASD(A_{2};B_{1},B_{2})\\ & =\;RAS(A_{1};B_{1})-RAS(A_{2};B_{1})\\ & \;-RAS(A_{1};B_{2})+RAS(A_{2};B_{2}) \end{array}$$

Those derived RASD-statistics can be used to test symmetry or treeness, similar to the widely used $F_{4}$ or *D* statistics (Patterson et al. 2012). $RASD(A_{1},A_{2};B)$ corresponds to $F_{4}(A_{1},A_{2};B,O)$, which tests relative sharing between $A_{1}$ and $A_{2}$ with respect to *B*. Similarly, $RASD(A;B_{1},B_{2})$ corresponds to $F_{4}(A,O;B_{1},B_{2})$. Ultimately, the difference of differences $RASD(A_{1},A_{2};B_{1},B_{2})$ corresponds to $F_{4}(A_{1},A_{2};B_{1},B_{2})$.

Note that this correspondence to $F_{4}$ statistics becomes an equivalence in the special case that all sites are non-missing in all considered groups, or only sites non-missing in all groups are considered (e.g. using maxmiss = 1 in the Software qpfstats from *ADMIXTOOLS2* (Maier et al. 2023), and no allele frequency ascertainment is applied (i.e. $f_{\min}=0$ and $f_{\max}=1$). In the more general case relevant here, due to different patterns of missing SNPs in ancient samples, our linear combinations in the definition of RASD are not strictly equal to $F_{4}$ even without frequency ascertainment.

### Ancestry decomposition

We here propose a method how to use RAS and RASD statistics to compute ancestry proportions in admixture scenarios. Specifically, we model a given (admixed) *target population T* as a linear sum of *source populations*  $\left\{S_{1},S_{2},\ldots ,S_{n}\right\}$, with the coefficients $\left\{\beta_{1},\beta_{2},\ldots ,\beta_{n}\right\}$ summing up to 1. Symbolically, we can write


(3) $$\begin{array}{cc} & T=\sum_{i=1}^{n}\beta_{i}S_{i}\sum_{i=1}^{n}\beta_{i}=1 \end{array}$$

The key idea is to represent the *target* and *sources* by their rare allele sharing (as estimated using RAS-statistics) with a selected group of *reference populations*. Following the nomenclature from the popular qpAdm Software (Patterson et al. 2012; Maier et al. 2023), we denote *target* and *sources* as *left populations*, and *references* as *right populations*.

Specifically, we choose a set of *m right populations*  $\left\{R_{1},R_{2},\ldots ,R_{m}\right\}$. If *T* is then admixed as specified in equation (3), and if there was no gene flow going from the *left* into the *right populations* (only from *right* to *left*, see below for a discussion on relaxing this condition), then we can write:


(4) $$\left(\begin{array}{c} RAS(T;R_{1})\\ RAS(T;R_{2})\\ \vdots \\ RAS(T;R_{m}) \end{array}\right)=\sum_{i}\beta_{i}\left(\begin{array}{c} RAS(S_{i};R_{1})\\ RAS(S_{i};R_{2})\\ \vdots \\ RAS(S_{i};R_{m}) \end{array}\right)$$

Defining the vectors $v=\{v_{j}\}=\{RAS(T;R_{j})\}$ and $b=\{\beta_{i}\}$, and the matrix $W=\{W_{ij}\}=\{RAS(S_{i};R_{j})\}$, we can write this as:


(5) $$v=W^{T}\cdot b$$

This would be a simple linear regression model, but we still have to satisfy the constraint $\sum_{i=1}^{n}\beta_{i}=1$. We therefore first write the last element of b as


$$\beta_{n}=1-\sum_{i=1}^{n-1}\beta_{i}$$

Restricting to a single row *j* of the equation, we then get


(6) $$v_{j}=\sum_{i=1}^{n-1}\beta_{i}W_{ij}+\left(1-\sum_{i=1}^{n-1}\beta_{i}\right)W_{nj}$$

which in turn becomes


(7) $$v_{j}-W_{nj}=\sum_{i=1}^{n-1}\beta_{i}(W_{ij}-W_{nj})$$

The differences on both sides of the equation are actually RASD-statistics. We define $X_{ij}=W_{ij}-W_{nj}=RASD(S_{i},S_{n};R_{j})$ for i=1,…,(n−1), $y_{j}=v_{j}-W_{nj}=RASD(T,S_{i};R_{j})$ and $a=\{\beta_{1},\ldots ,\beta_{n-1}\}$ can then write


(8) $$y=X^{T}\cdot a$$

or


(9) $$\left(\begin{array}{c} RASD(T,S_{n};R_{1})\\ RASD(T,S_{n};R_{2})\\ \vdots \\ RASD(T,S_{n};R_{m}) \end{array}\right)=\sum_{i=1}^{n-1}\beta_{i}\left(\begin{array}{c} RASD(S_{i},S_{n};R_{1})\\ RASD(S_{i},S_{n};R_{2})\\ \vdots \\ RASD(S_{i},S_{n};R_{m}) \end{array}\right)$$

which is a simple linear regression equation. The least-square solution of this equation is (see Hastie et al. 2009):


(10) $$\hat{a}=(XX^{T}{)}^{-1}Xy$$

which is a genome-wide point-estimate of admixture proportions. There are two sources of uncertainty/error to consider in this estimation: First, the sampling noise and the finite length of the genome, and second the standard error from the least-square fit itself. We estimate both of these errors using a genome-wide block-Jackknife procedure (Busing et al. 1999) to re-estimate the admixture proportions using equation (10) with each of the blocks removed, and derive the standard error of them. We highlight a difference to qpAdm Haak et al. (2015), in that we do not explicitly capture covariance between elements of the RASD-matrix here, but rely on the overall Jackknife procedure to capture effects of these covariances on the final estimate.

The basic decomposition (equation (4)) relies on there being no gene flow from left to right populations after admixture of the target formed, only vice versa. The argument for this assumption follows an argument originating in Reich et al. (2012) and refined in Haak et al. (2015) and it underlies the basic linear algebra of a matrix of left and right population $F_{4}$ statistics. See also Williams and Huber (2025) for a more detailed investigation of this condition. We posit that the same argument holds for *RAS*, as long as ascertainment on frequencies is exclusively based on right populations. In fact, within the context of this argument, there is no fundamental difference between *RASD* statistics and $F_{4}$ statistics. The machinery behind qpAdm is agnostic of the specific type of ascertainment, as long as it occurs outside of the set of left populations.

Absence of such left-to-right gene flow after a target formed is hardly ever true in real populations. However, in contrast to ordinary *F*-Statistics, which is the basis for ancestry decomposition in qpAdm, *RAS* values are only affected by reverse gene flow if it occurred very recently. This can often be ruled out. But even if not, violations of the no-left-to-right gene flow assumption may affect goodness-of-fit statistics more than the actual ancestry estimates which we focus on here.

### Implementation of the method

All scripts used to process and analyze data, exclusively in the R programming language (Ihaka and Gentleman 1996), are provided within a GitHub repository https://github.com/huanglei-artificium/RAS_tools, including documentation.

Briefly, our tool computes RAS and RASD with flexible ascertainment on allele frequency. Besides .geno files, our tool also accepts allele frequency data consisting of two columns representing numerator (nr of alternative alleles) and denominator (nr of non-missing haplotypes) as input. Then we compute allele frequencies for each population and each site, which are then used to i) select sites that fulfill ascertainment conditions, and ii) to compute the actual RAS-statistics on those sites.

To compute optional uncertainties based on a blockwise jackknife estimate (Busing et al. 1999), RAS gets computed block-wise (typically by chromosome), which are then combined to obtain genome-wide statistical values.

Multiple statistics, cycling through several populations, and multiple ascertainment conditions are handled efficiently inside our tools, and can be computed in one run.

### Simulations

**Fig. 1.**
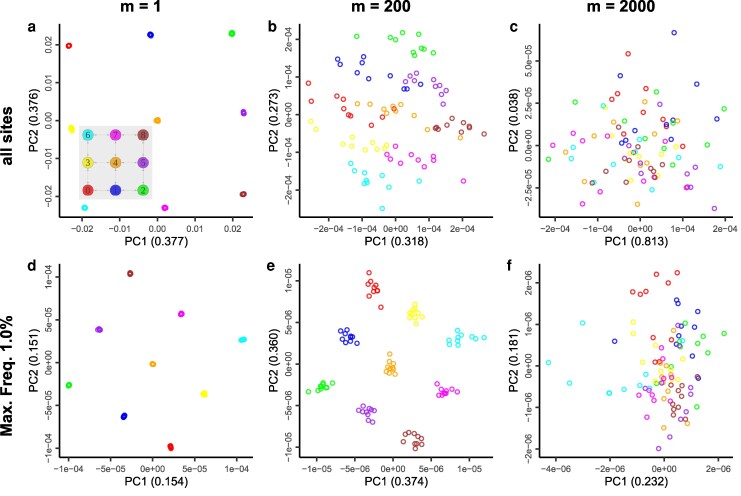
The first two principal components (PC1 and PC2) of the nine-dimensional dataset $\left\{F_{3}(O;x,R_{i})\right\}$ a), b) and c) and $\left\{RAS(x,R_{i})\right\}$ d), e) and f) on test individuals *x* and nine reference populations ($R_{0}$ to $R_{8}$) at different migration rates. The values in parentheses represent the variance proportions of the PC dimension. The SNP panels used are all sites a) b) and c) and sites with derived allele frequency less than 1% in all reference individuals (D, E and F). The migration rates (*m*) used in the simulation are 1 a) and d), 200 b) and e) and 2000 c) and f). Test individuals *x* are distinguished by colors representing different populations, shown in legend in a), which also includes the schematic of simulated population migration with gray background.

To illustrate how RAS and RASD perform in contrast to $F_{3}$ and $F_{4}$ statistics, we devised a simulation scheme that allows for varying levels of population structure by tuning migration rates. Specifically, we use msprime (Kelleher et al. 2016) to simulate a set of nine populations, located in a 3×3 grid, numbered from 0 to 8 following a left-to-right, bottom-to-top order, as depicted in Fig. 1a. Each population has an effective diploid population size $N_{e}=20,000$, with n=50 diploid individuals sampled from each population. Each population is connected to its non-diagonal neighboring populations through an equal and symmetric two-way per-generation migration rate. The migration rate is altered across different simulations to produce different degrees of population structure among the simulated populations. We followed three temporal scenarios: (i) a constant scaled migration rate ($m=4m_{0}N_{e}$) varying across m={1,2,5,10,20,50,100,200,500,1000,2000,5000} respectively; (ii) a scenario mimicking recent mixing of diverged lineages, setting a low early-stage scaled migration rate $M=4M_{0}N_{e}=1$ for t>50 generations ago, and a late-stage scaled migration rate $m=4m_{0}N_{e}$ for 0<t≤50 generations ago varying across to m={1,5,10,50,100,500,1000,5000} respectively; (iii) a scenario mimicking recent divergence, with the late-stage scaled migration rate fixed at a low rate of m=1 for 0<t≤50 generations ago, and the early-stage scaled migration rate *M* for t>50 generations ago varying across to M={1,5,10,50,100,500,1000,5000} respectively. Each sample has 20 chromosomes, each 100Mbp in length. No specific outgroup was simulated, as in our simulations the ancestral allele is known by construction.

To assess the population structure in the simulated data, we perform the statistical analysis across three different ascertainment schemes (i) Using all variants in the simulated data, (ii) Using only rare variants with different ascertainment conditions, (iii) Using a random subset of 1.2M variants with derived allele frequency between 0.05 and 0.95, to mimic the 1240K panel (Mathieson et al. 2015).

We chose 10 individuals from each population to act as “test individuals” (denoted by $T_{i}$), whereas the other 40 individuals are then used as References $R_{i}$. The ascertainment is therefore based on 9×40=360 reference individuals. For some analysis, in order to average sampling noise, we rotated test and reference individuals 5 times so that each individual served as a test individual once. The code for running the simulation is available at https://github.com/Schiffels-Popgen/RAS_exploration.

### Modern reference data

For our modern reference data, we chose as a starting point the recently released harmonized dataset of 1,000 Genomes Project (1kGP) and Human Genome Diversity Project (HGDP) (Koenig et al. 2024 ), where a new genotype calling was made based on the raw sequencing data from 1kGP and HGDP, with more than 150 million high-quality variants identified, including a large number of rare variants. We chose to focus on the European populations in this dataset, which includes five from 1kGP and eight from HGDP, in which genetic outliers and relatives closer than second-degree were filtered out according to the analysis of Koenig et al. (2024) (see Table 1 for the number of individuals for each population). We further supplemented this basic dataset with three European public datasets with genome-wide allele count data: Danish from GenomeDenmark project (Maretty et al. 2017), Dutch from Genome of the Netherlands (GoNL) project (The Genome of the Netherlands Consortium 2014) and Swedish from SweGen project (Ameur et al. 2017) (Table 1).

**Table 1.** Number of haploid copies (2N) of present-day populations used for analysis.

Population abbr.^a^	Population	Haploids	Dataset
CEU	Northern and Western European ancestry	242	1 kGP
FIN	Finnish	196	1 kGP
GBR	British	176	1 kGP
IBS	Spanish	208	1 kGP
TSI	Toscani in Italy	206	1 kGP
-	Adygei	34	HGDP
-	Basque	46	HGDP
-	French	54	HGDP
-	Italian	22	HGDP
-	Orcadian	26	HGDP
-	Russian	50	HGDP
-	Sardinian	54	HGDP
-	Tuscan	16	HGDP
DK	Danish	40	GenomeDenmark
NL	Dutch	998	GoNL
SE	Swedish	2,000	SweGen
AFR_all	All Africans	1,978	1kGP+HGDP

^a^Where available, we use abbreviations in the figures.

Even though we focused on Europe, for all the RAS statistics regarding real data, we used all African groups in 1kGP and HGDP as outgroups, ascertaining to strictly fixed sites within Africa.

We screened 133 million biallelic SNPs from the harmonized dataset of 1kGP and HGDP for analysis. Variant sites in the Danish, Dutch and Swedish datasets were filtered to this 1kGP+HGDP SNP set, to ensure that our African outgroup is available on all analyzed SNPs. We excluded sites with different alternative alleles when joining the datasets. Unless otherwise stated, we used all European populations in Table 1 for references, which consist of 16 populations and 2,184 individuals (4,368 sets of haploid chromosomes). Allele frequencies for each site are based on all non-missing individuals.

### Ancient genomes

Our ancient dataset consists of 34 individuals from Great Britain with shotgun-sequencing data, 7 dating to the Late Iron Age (LIA) and 27 to the Early Middle Ages (EMA) (Martiniano et al. 2016; Schiffels et al. 2016; Gretzinger et al. 2022). We started with alignment files (.bam files) and called variants overlapping with our reference SNPs, using the Majority-Call method with a minimum coverage of 3 and downsampling in pileupCaller (article submitted). We then selected individuals with more than 1 million SNPs overlapping with our reference data set (see above). We used the ancestry decomposition published in Gretzinger et al. (2022), with two major components: CNE (“Continental North European”) and WBI (“Western British-Irish”) clearly separated by a high-resolution PCA with thousands of individuals, and labeled individuals whose dominant ancestry (CNE or WBI) is more than 70% as “England_CNE” (N=17) and “England_WBI” (N=7).

## Results

### Exploring RAS with simulated data

We first explored, how our RAS-Statistics can distinguish populations from our simulated 3×3 grid of connected populations (Methods). For all test individuals *x* across all populations, we computed $RAS(x;R_{i})$, choosing each of the nine populations as reference (namely from $R_{0}$ to $R_{8}$), and calculated principal components of the dataset $\left\{RAS(x,R_{i})\right\}$, effectively projecting a 9-dimensional space to two-dimensions. For comparison, we also computed PCA based on $F_{3}(O;x,R_{i})$, without any allele frequency ascertainment.

In the constant migration scenario, for low migration rate, both RAS and $F_{3}$ reveal clearly separated clusters of individuals, corresponding to the 9 populations (Fig. 1a). Increasing the migration rate shows less clearly defined clusters in the case of $F_{3}$, with no apparent structure being visible for the highest migration rate tested here (m=2,000) (Fig. 1b and c). In contrast, ascertaining SNPs to be rare with respect to the reference populations reveals structure being visible also at higher migration rates. For example, at m=200 (Fig. 1e), RAS scatter plots reveal still well-separated clusters while $F_{3}$ (Fig. 1b) already shows considerable overlap between groups. Even at m=2000 (Fig. 1f), RAS still shows some power to distinguish groups, whereas $F_{3}$ appears random (Fig. 1c). For our simulated “1240K” dataset with only 1.2 million common variants, structure is substantially less resolved (Supplementary Fig. S1). Finally, with the highest simulated migration rate, even rare variation appears quite random (Supplementary Fig. S1).

Following this qualitative assessment of the ability to separate closely related groups, we devised a more quantitative assessment, by testing for each individual whether it is closest to the mean position (using the first two principal components) of their own population or to some other population, in which case we consider it misclassified. The misclassification ratio is then the proportion of misclassified test individuals relative to all test individuals (using the rotation scheme described in Methods, this amounts to 450 tests). In the constant migration rate scenario, as expected RAS performs better in distinguishing populations for medium and high migration rates, although at the highest migration rate m=5000, the improvement is relatively weak (Supplementary Fig. S2A). Specifically, at m=200,500  RAS can still distinguish the test individuals with no or little error, while $F_{3}$ statistics (for both “all sites” and 1,240 K) start to misclassify.

In the scenario of recent structure (with recent migration rates being low after earlier mixing, see Methods), we find that RAS again outperforms $F_{3}$-based PCA. This is expected, as RAS specifically focuses more on recent structure (Supplementary Fig. S2C). As expected, in the opposite case, where deeply diverged lineages recently mixed, RAS does not convey an advantage over $F_{3}$ (Supplementary Fig. S2B).

### Application to real data

**Fig. 2.**
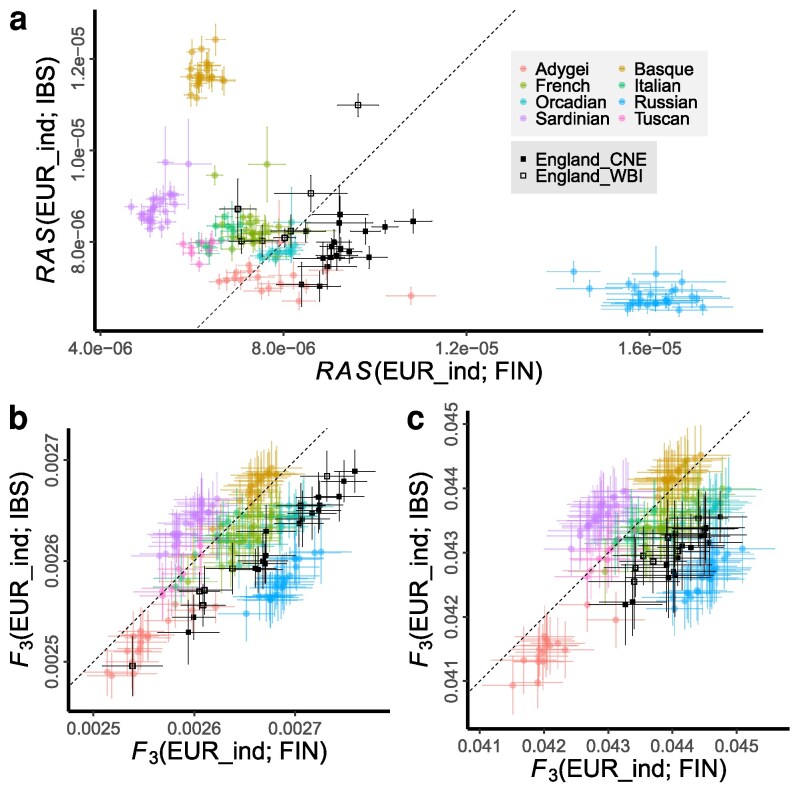
RAS and outgroup-$F_{3}$ statistics on European individuals (*x*) and FIN/IBS, shown with error bars of ±1 standard deviation (SD). Modern European individuals are distinguished by colors representing different populations in HGDP; ancient British individuals are marked black and distinguished by shapes representing different ancestries. SNP panels are based on the harmonized dataset of 1kGP and HGDP with the following filtering: monomorphic in all 1kGP Africans and derived allele frequency less than 2% in all 1kGP Europeans a); all sites b); 1240K sites c). The dashed line indicates equal relationship to FIN and IBS. FIN: Finnish; IBS: Spanish.

Turning to real data, we first analyzed present-day European genetic diversity. Specifically, we used the five European populations from the 1,000 Genomes Project (1kGP) as references (The 1000 Genomes Project 2015) and eight European populations from the HGDP project as test individuals (Bergström et al. 2020). We then quantified the affinity between 1kGP European populations and each European HGDP individual with outgroup-$F_{3}$ and RAS (Fig. 2). FIN (Finnish) and IBS (Spanish) from 1KGP are selected as references because they are relatively different geographically and genetically. Both whole genome and 1240K SNP sets reveal differential affinities of the HGDP populations with respect to these references (Fig. 2b and c). For example, Russian and Sardinian groups are closest to FIN or IBS references, respectively. Ascertaining on rare allele frequencies in the references with RAS, these differences become substantially larger (Fig. 2a). In particular, Russian and Basque groups from the HGDP share substantially more rare alleles with either FIN or IBS, indicating recent shared ancestry between Russian and Finnish, and between Basque and Spanish. In addition, there are clearer boundaries for some isolated populations, such as Sardinian and Orcadian. All groups are more clearly separated with RAS than with un-ascertained variants.

We next turned to ancient DNA to explore the potential of rare variants to analyze ancient population structure. Specifically, we again used the present-day 1kGP reference groups and measured RAS and $F_{3}$ to a set of ancient genomes from England (Gretzinger et al. 2022) for which whole-genome sequencing data is available. Without ascertainment, these two groups do not appear to separate clearly on either all sites or 1240K subset (Fig. 2b,c). In particular, WBI individuals are distributed among the entire range of CNE individuals. In contrast, we find that in our RAS analysis (Fig. 2a), the WBI individuals fall closer to present-day French compared to samples with CNE ancestry, with all WBI individuals being closer to IBS than to FIN, as indicated by the dashed line in Fig. 2a.

### Testing for population structure with RASD

As a formal test of population structure we can test for symmetry between two groups with respect to a reference group. For classical *F*-Statistics, this is done through $F_{4}$-Statistics, which are essentially differences of $F_{3}$-Statistics, to statistically quantify differential affinities as deviations from symmetry. Analogously, we defined $RASD(A_{1},A_{2};B_{1},B_{2})$-Statistics (Methods), which tests whether $A_{1}$ and $A_{2}$ are differentially related to $B_{1}$ and $B_{2}$.

**Fig. 3.**
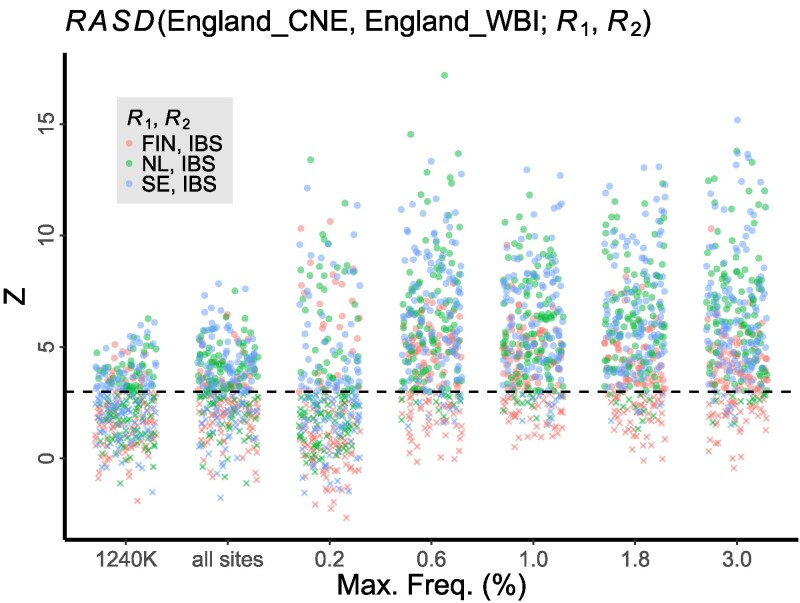
Z score distribution of $RASD(\text{England}\_\mathrm{CNE},\text{England}\_\mathrm{WBI};R_{1},R_{2})$ using different ascertainment schemes shown along the *x* axis. Results are shown with horizontal jitter to avoid overplotting. The ascertained cases are represented in numbers, which are $p_{\max}$ (in percent) in 1kGP and HGDP European populations plus the Dutch, Danish, and Swedish populations. Different colors represent different reference population pairs $\left(R_{1},R_{2}\right)$. Results with Z<3 are indicated as crosses and with Z>3 with circles.

Gretzinger et al. (2022) showed that CNE and WBI ancestries are genetically differentiated based on a high-resolution PCA by projecting ancient British individuals onto thousands of modern European individuals. In order to reproduce this using *RASD* and with far fewer samples, here we explore the following form: $RASD(\text{England}\_\mathrm{CNE},\text{England}\_\mathrm{WBI};R_{1},R_{2})$, where the first two slots are cycling through ancient individuals from Great Britain with dominant CNE (n=17) or WBI (n=7) ancestry respectively (Methods), and the last two slots are cycling through various present-day reference populations. We evaluated the results using the Z-Score (i.e. the statistical deviation from zero), using RASD at different ascertainment conditions and corresponding $F_{4}$ at 1240K and all sites (Fig. 3). We find that RASD with maximum allele frequencies ascertained to between 0.6 and 3 percent can better distinguish between CNE and WBI compared to $F_{4}$, with Z scores even greater than 10 for some combinations. When lowering the allele frequency cutoff to 0.2%, power decreases because the number of analyzed positions drops too far, increasing noise. Among the tested reference populations, NL (Dutch) and SE (Swedish) resulted in better discrimination than pairs involving FIN (Finnish). This is consistent with immigrants into Britain from continental Europe during Early Middle Age being genetically closer to present-day Dutch or Swedish Gretzinger et al. (2022).

**Fig. 4.**
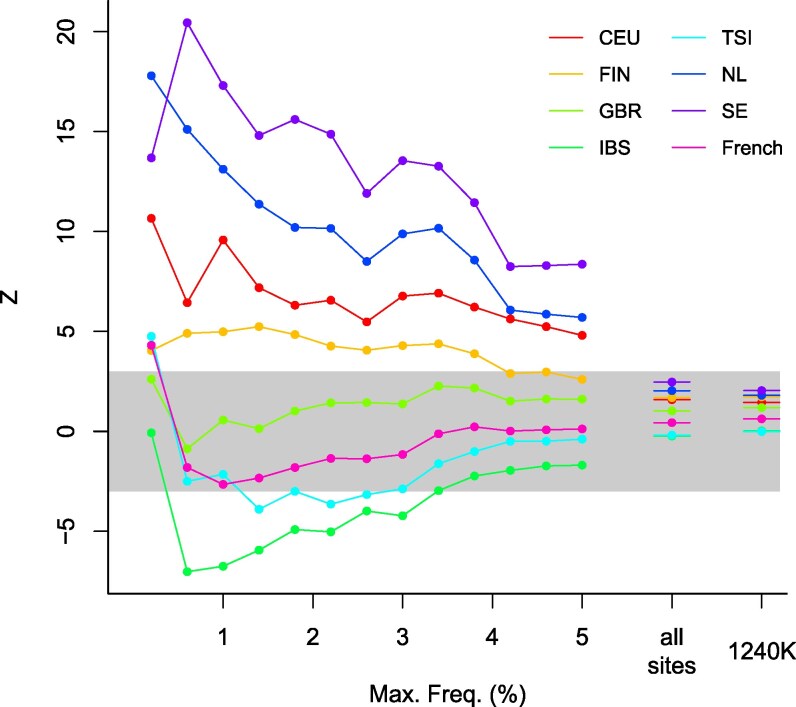
Distinguishing CNE and WBI ancestry using RASD and $F_{4}$ represented in Z scores. The colored lines are Z scores of RASD(England_CNE,England_WBI;R), where *R* is a selected present-day European population, under different $p_{\max}$ (in percent) in present-day European references. Two categories on the right cover ordinary $F_{4}(\text{England}\_\mathrm{CNE},\text{England}\_\mathrm{WBI};R,\text{AFR}\_\mathrm{all})$ on all sites and on 1240K, respectively. The gray area represents |Z|<3.

In order to explore the genetic change in Early Middle Age Britain, we performed a systematic analysis by grouping individuals with CNE (n=17) and WBI (n=7) ancestry respectively and comparing them with present-day European populations, under different ascertainment conditions (Fig. 4).

For the 1240K panel and all sites, we observe non-significant positive Z scores for all European reference populations (Fig. 4 on the right). In contrast, with RASD-statistics, the difference between CNE and WBI, represented by Z score, becomes much more pronounced at low derived allele frequency. Among the present-day European populations, SE (Swedish), NL (Dutch), CEU (Northern and Western European ancestry) and FIN (Finnish) have better ability to distinguish CNE and WBI than others, suggesting that these present-day populations are most closely related to the actual source that migrated into early medieval Britain, consistent with previous conclusion. (Gretzinger et al. 2022).

Rare alleles can provide information about population history at different points in the past, which is reflected by the results of RASD-statistics at different derived allele frequency cutoffs. For French, TSI and IBS, their Z scores are higher at very low frequency 0–0.2% than at 0–0.6%, which is due to recent low-level gene flow within the European continent making present-day Southern Europeans share more such rare alleles with CNE individuals than with WBI individuals. The Z scores of French and TSI at 0–0.2% are even slightly higher than those of FIN, reflecting a geographic pattern of this recent continental gene flow, since Finnish and the estimated location of CNE ancestry are on the opposite sides of the Baltic Sea. However, the Z score of FIN reaches its peak at maximum cutoff 1.4% and still remains significant until maximum cutoff 3.8%, suggesting that the gene flow between the CNE ancestry and the ancestors of Finnish occurred in the more distant past, probably mediated through a population closely related to present-day Swedish.

**Fig. 5.**
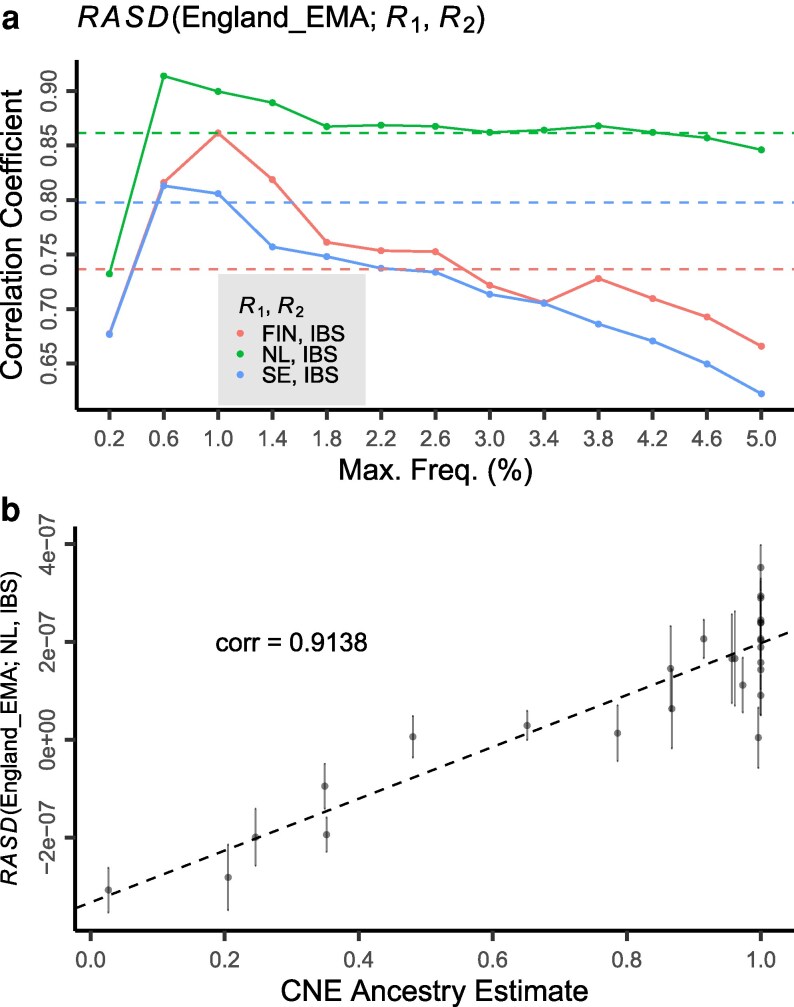
a) The colored lines are the correlations between $RASD(\text{England}\_\mathrm{EMA};R_{1},R_{2})$ and estimates of CNE ancestry of each England_EMA individual (from Gretzinger et al. 2022), under different ascertainment frequency $p_{\max}$ (in percent) in present-day Europeans. The dashed lines are the correlations of corresponding $F_{4}$-statistics on 1240K. b) RASD(England_EMA; NL, IBS) with error bars of ±1 SD, ascertained on sites with 0–0.6% derived allele frequency in present-day Europeans.

So far, we have considered those ancient British individuals with predominantly either CNE or WBI ancestry. However, actual CNE admixture in EMA Britain formed a spectrum with varying proportions among different individuals (Gretzinger et al. 2022). As *F*-statistics are linear under a gradient of admixture components, so are RAS and RASD. To explore this, we computed RASD and *F*-statistics on each EMA British individual and a pair of present-day European reference populations that are able to distinguish EMA British individuals. We then computed the correlation between our estimates and the high-resolution CNE ancestry estimated by supervised admixture using thousands of present-day Europeans as reported in Gretzinger et al. (2022) (Fig. 5; Supplementary Fig. S3). At specific $f_{\max}$, such as 0.6% and 1%, our RASD estimates using any of Finnish (FIN), Netherlands (NL) or Swedish (SE), together with Spanish (IBS), show a higher correlation to the reported CNE ancestry estimates than $F_{4}$-values on 1240K, indicating a better resolution in distinguishing EMA British individuals with rare alleles, and potentially a more accurate estimation on CNE ancestry from new samples provided the RASD statistical values.

### Decomposing ancestries using linear combinations of RAS

Motivated by the correlation between RAS-Statistics and ancestry components, we devised a new method to decompose ancestry components based on RAS (see Methods).

Briefly, every left population (i.e. the target and the sources) has a specific profile of rare allele sharing with each of the right populations, represented by a multi-dimensional vector. We then model the target profile as a linear combination of source profiles, with the coefficient reflecting the admixture proportion.

We tested this new method on our simulated isolation-migration model (Method), with the populations arranged as a 3×3 grid and centrally symmetric about population 4. Although the simulation scheme is not based on explicit “admixture” but continuous migration, due to its symmetry, population 4 can be considered being “50%/50% admixed” between two populations that are centrally symmetric about it.

Specifically, we model population 4 as being admixed between population 0 (bottom-left) and population 8 (top-right), and within this model space expect the correct result to be 0.5 for each. In order to evaluate our ancestry estimates based on RAS, we defined two types of error: (A) the absolute difference between our estimate and 0.5, denoted as “true error”; (B) the standard error of our estimate based on a chromosome-wise jackknife (Busing et al. 1999), denoted as “standard error”.

**Fig. 6.**
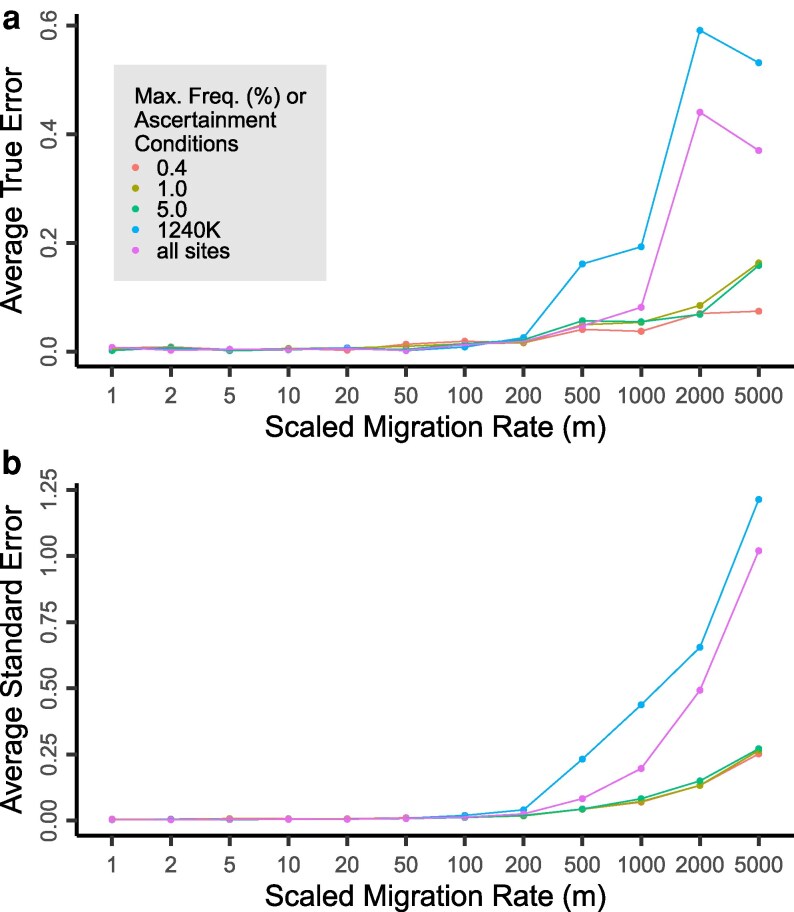
Error estimate for modeling population 4 as an “admixture” of population 0 and 8. Different colors represent different ascertainment conditions $p_{\max}$ (in percent), common sites and all sites. We rotated test individuals for 5 times and calculated the average of errors for 5 parallel tests.

At high migration rates, both types of errors are substantially lower for our RAS-based ancestry estimate compared to $F_{3}$-based estimates on 1240K or even on all sites (Fig. 6). Towards lower migration rates, we observe a subtle turning point (around m=100, Supplementary Fig. S4), where all sites and even 1240K are performing subtly better than rare variants, although at a very low level of error, which we attribute to a lower number of shared rare variants for low migration rates. Therefore in real cases, it is appropriate to use *F*-statistics when populations are highly differentiated, while RAS fills the gap where *F*-statistics lose resolution.

## Discussion

We have defined RAS, a statistical method based on rare allele sharing between populations, and demonstrated that rare variation provides powerful means of identifying fine-scale population structure and revealing unique population histories that common alleles may not capture. For both simulated and empirical data, we observe a signal reinforcement of recent demographic events, reflected in the much stronger allele sharing for rare alleles (Figs. 1d,e,f; 2a; 3; 4), compared to common alleles and even the whole genome.

Insights into population structure obtained through population genetic analyses have greatly advanced our understanding of historical human populations, and their migration and admixture (Nielsen et al. 2017; Skoglund and Mathieson 2018; Liu et al. 2021; Stoneking et al. 2023). Especially in recent years, studies involving larger sample sizes have revealed more detailed historical demographic events (Lazaridis et al. 2022; Allentoft et al. 2024; Antonio et al. 2024; McColl et al. 2025a). Here, we show that beyond increasing sample sizes, expanding the analyses towards rare variants can provide a view on especially recent population structure with high resolution, such as the clearly demarcated Russian and Basque populations (Fig. 2a). Even for ancient DNA, by embracing a rare allele ascertainment scheme strictly in present-day data, we are able to see the significantly improved discrimination between populations, as exemplified here by differentiating between CNE and WBI ancestries (Figs. 3; 4; 5). Surprisingly, the resolution for rare variants is even higher than using all variants (Figs. 2; 3), which further emphasizes the importance of ascertaining rare alleles for population structure analysis.

The role of this ascertainment based on allele frequency is very different from the role of outgroup- or heterozygosity-based ascertainment in array of SNP enrichment panels. With the latter type of ascertainment, one expects to be able to approximate genome-wide results based on the entire demographic history of a set of populations. A critical question then is whether the respective ascertainment provides biases in the statistics based on the ascertainment SNPs compared to whole-genome estimates Patterson et al. (2012) and Flegontov et al. (2023). In contrast, constraining allele frequencies does not attempt to approximate genome-wide population history, but provides an indirect constraint on allele ages. Since recent demographic events are usually different from the overall demographic history, we in fact expect different results (and population structures as reflected in those results) between RAS and *F*-statistics, and among different ascertainments of RAS-statistics.

We have restricted our RAS analysis of ancient DNA to samples with available whole-genome (shotgun-) data. While we have experimented with using capture data, relying on the few additional rare variants that might get covered due off-target sequencing reads, we have not found this to be sufficient. Fortunately, recent advances in sequencing technology have made shotgun sequencing more efficient and cost-effective, and shotgun data are becoming increasingly available for ancient DNA studies (Allentoft et al. 2015; Maisano Delser et al. 2021; Allentoft et al. 2024; Mallick et al. 2024; McColl et al. 2025a, 2025b; Speidel et al. 2025), transforming the field of archaeogenetics as a whole by offering more detailed insights into human population history.

In western Eurasia this development is particularly promising: Through the spread of early European farmers from the Near East (Lazaridis et al. 2014), and the movement of Indo-European speaking groups from the Eurasian steppe (Haak et al. 2015), populations that might have been more genetically distinct have gradually become more similar, leading to an overall more homogeneous genetic structure across Europe, which may have remained stable since the Iron Age (Antonio et al. 2024). Nonetheless, regional differences still persist, which are shaped by local history: during the Roman period, while Northern provinces maintained higher levels of local continuity, Southern sites absorbed the influences from Northern Africa, the Near East, and Eastern European Slavic groups, displaying more genetic variability (Antonio et al. 2019; Olalde et al. 2023); Celtic and Germanic tribes occupying different regions of Europe had different genetic profiles due to their different migration routes and interactions with different neighboring groups (McColl et al. 2025a). Substantial progress on these question reflects novel method developments based on haplotype based analysis, such as IBD-based inference (McColl et al. 2025a) or ancestral recombination graph inference (Speidel et al. 2025). Here, using RAS, we offer an additional tool that may in some contexts yield additional insights (e.g. among recently diverged, closely related populations), while being computationally simpler.

In our final demonstration, we have implemented a new way of estimating ancestry proportions, showing a better performance of RAS-based compared to ordinary *F*-statistics-based estimates (Fig. 6). In the framework of *F*-statistics, there are extensions based on $F_{4}$ matrices: qpWave for testing symmetry or external sources, and qpAdm for testing hypothetical admixture modeling (Patterson et al. 2012). Left for future work, RAS-statistics may be used similarly to develop formal tests for symmetry and admixture.

Larger sample sizes have a more direct impact on the resolution of our method compared to most traditional tools based on common alleles such as *F*-statistics and PCA. Since rare alleles occur at low frequencies, their frequency estimates lead to greater relative error than those of common alleles. Increasing sample size therefore plays a particularly important role in reducing this error, improving the accuracy of rare allele frequency estimates and, in turn, enhancing the resolution of RAS statistics. With sequencing prices and ancient DNA technology continuing to improve, the methods presented here will become more powerful over time.

## Supplementary Material

jkag100_Supplementary_Data

## Data Availability

All data used in this manuscript can be accessed from public resources. All modern genomes or allele frequency data (.vcf files) can be obtained from the following websites: the harmonized 1kGP+HGDP dataset at https://gnomad.broadinstitute.org/downloads#v3-hgdp-1kg (Koenig et al. 2024), allele frequency data from the GenomeDenmark project at https://ega-archive.org/studies/EGAS00001002108 (Maretty et al. 2017), allele frequency data from the Genome of the Netherlands (GoNL) project at https://www.nlgenome.nl/menu/main/app-go-nl/download-data (The Genome of the Netherlands Consortium 2014), allele frequency data from the SweGen project at https://ega-archive.org/studies/EGAS50000000906 (Ameur et al. 2017). All ancient genomic data from Great Britain (.bam files or raw read data from the European Nucleotide Archive) from Martiniano et al. (2016); Schiffels et al. (2016) ; Gretzinger et al. (2022) as described in these publications. Supplemental material available at G3 online.

## Code availability

Software to estimate RAS, RASD and ancestry decomposition, as well as to prepare the datasets, can be found at https://github.com/huanglei-artificium/RAS_tools. Documentation on how to use the software is available in the accompanying README file. The scripts used for data simulation are available at https://github.com/Schiffels-Popgen/RAS_exploration.
