# Supplementary material for: High-resolution analysis of recent population structure using rare variants
Source: G3 (Bethesda). 2026 Apr 24;16(6):jkag100. doi: 10.1093/g3journal/jkag100 (PMC13365844; doi:10.1093/g3journal/jkag100)
Supplement: jkag100_Supplementary_Data [file jkag100_supplementary_data.zip › Supplementary_Figure_Captions_G3-2026-406632.pdf]

Supplementary Figure Captions

Supplementary Figure S1 : The first two principal components (PC1 and PC2) of the nine-dimensional dataset $\{F_3(O; x, R_i)\}$ (A, B and C) on test individuals x and nine reference populations (R_0 to R_8) at different migration rates. The values in parentheses represent the variance proportions of the PC dimension. The SNP panels used are 1240K (A, B and C). The migration rates (m) used in the simulation are 1 (A), 200 (B) and 2000 (C). Test individuals x are distinguished by colors representing different populations, shown in legend in Figure 1A, which also includes the schematic of simulated population migration.

Supplementary Figure S2 : The misclassification ratio on the first two PC dimensions, with (A) a constant scaled migration rate ($m = 4m_0N_e$); (B) a fixed low early-stage scaled migration rate of $M = 4M_0N_e = 1$ for $t > 50$ generations ago, and a varying late-stage scaled migration rate $m = 4m_0N_e$ for $0 < t \leq 50$ generations ago; (C) a fixed low late-stage scaled migration rate of $m = 1$ for $0 < t \leq 50$ generations ago, and a varying early-stage scaled migration rate M for $t > 50$ generations ago.

Supplementary Figure S3 $RASD$(England_EMA; FIN, IBS) (A) and $RASD$(England_EMA; SE, IBS) (B) with error bars of ±1 SD, ascertained on sites with $0 - 0.6\%$ derived allele frequency in present-day Europeans.

Supplementary Figure S4 : Error estimate for modeling population 4 as an "admixture" of population 0 and 8. Different colors represent different ascertainment conditions including different p_{max} (in percent), common sites and all sites. We rotated test individuals for 5 times did 5 parallel tests.
