# Supplementary figures and images for: High-resolution analysis of recent population structure using rare variants
Source: G3 (Bethesda). 2026 Apr 24;16(6):jkag100. doi: 10.1093/g3journal/jkag100 (PMC13365844; doi:10.1093/g3journal/jkag100)

1240K

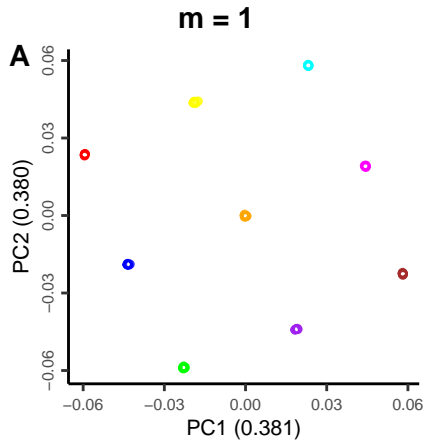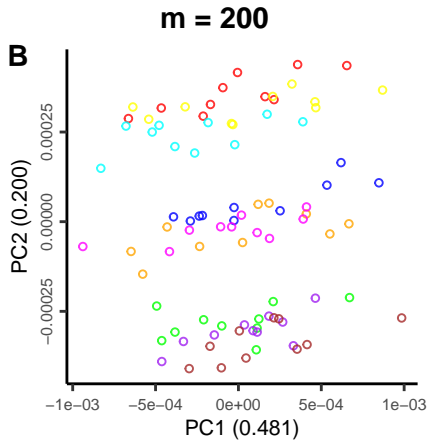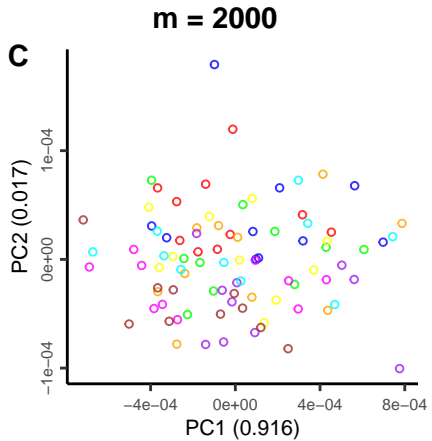

Supplement: jkag100_Supplementary_Data [file jkag100_supplementary_data.zip › Supplementary_Figure_S1_G3-2026-406632.pdf]

**A**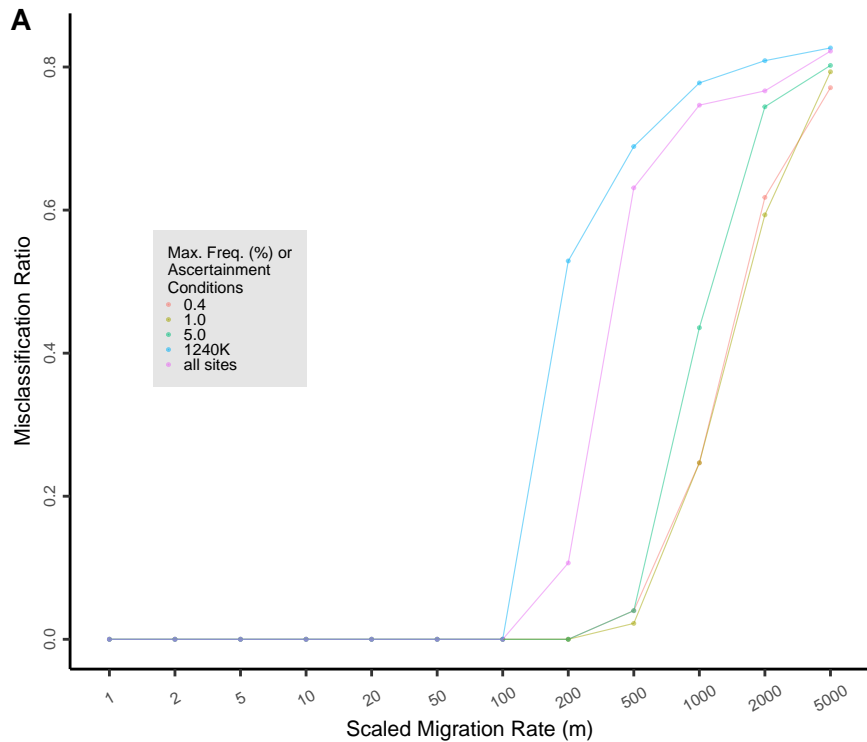**B**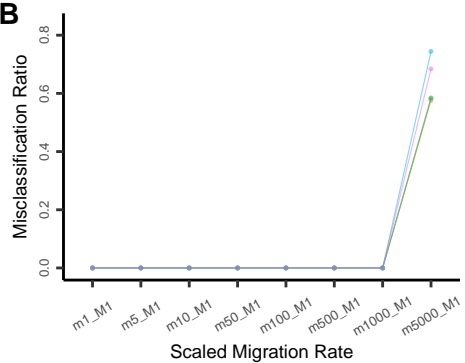**C**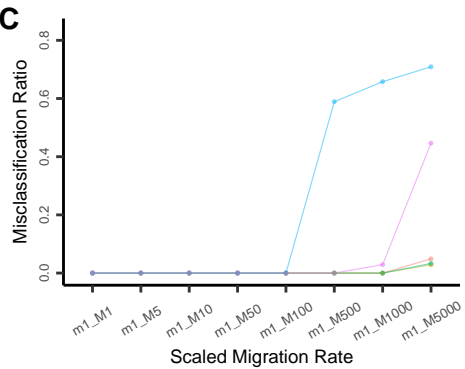

Supplement: jkag100_Supplementary_Data [file jkag100_supplementary_data.zip › Supplementary_Figure_S2_G3-2026-406632.pdf]

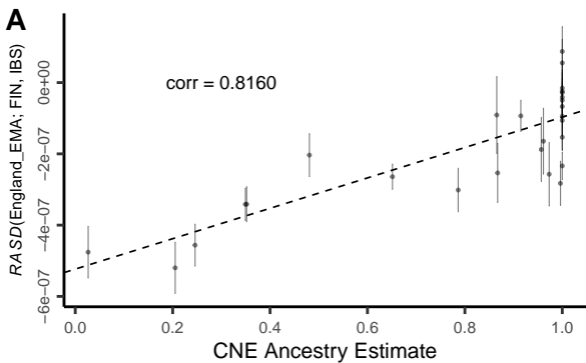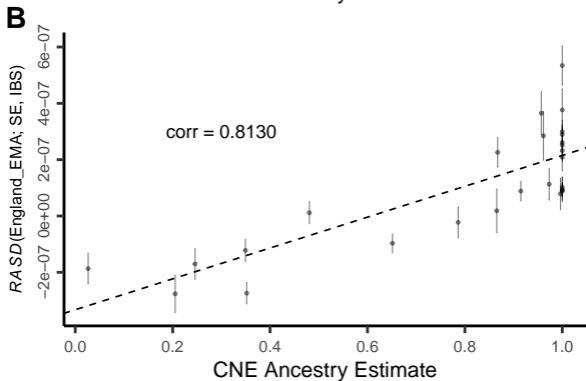

Supplement: jkag100_Supplementary_Data [file jkag100_supplementary_data.zip › Supplementary_Figure_S3_G3-2026-406632.pdf]

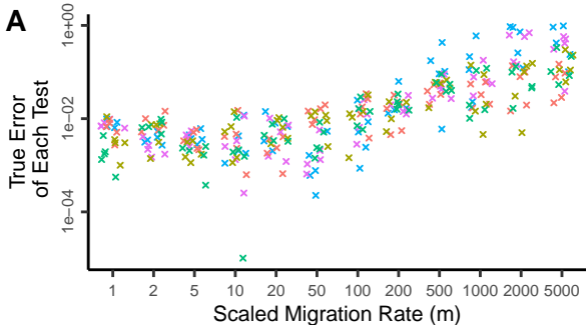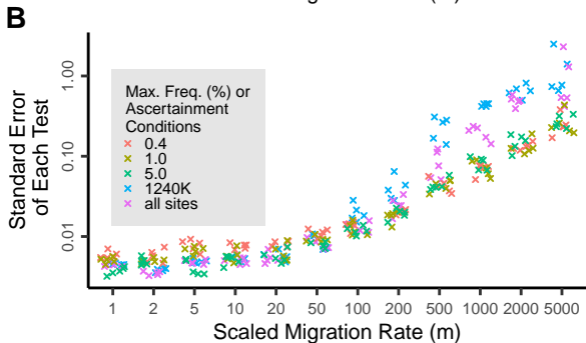

Supplement: jkag100_Supplementary_Data [file jkag100_supplementary_data.zip › Supplementary_Figure_S4_G3-2026-406632.pdf]
